# Socio-Economic Determinants of the Need for Dental Care in Adults

**DOI:** 10.1371/journal.pone.0158842

**Published:** 2016-07-21

**Authors:** Gilda Trohel, Valérie Bertaud-Gounot, Marion Soler, Pierre Chauvin, Olivier Grimaud

**Affiliations:** 1 U1099, INSERM, Rennes, France; 2 LTSI, University of Rennes 1, Rennes, France; 3 Dental Surgery Department, University Hospital, Rennes, France; 4 Research Team on Social Determinants of Health and Use of Care, UMRS 707, INSERM Paris, Paris, France; 5 Epidemiology Bio-Statistics Department, EHESP, Rennes, France; University of Washington, UNITED STATES

## Abstract

**Background:**

Oral health has improved in France. However, there are still inequalities related to the socio-economic status.

**Objectives:**

The aim of this study was to measure the prevalence of dental care needs in an adult population and to identify the demographic, socio-economic and behavioral variables that may explain variations in this parameter.

**Methods:**

A cross-sectional analysis of the French SIRS cohort (n = 2,997 adults from the Paris region; 2010 data) was carried out to determine the prevalence of self-reported dental care needs relative to demographic, socio-economic and behavioral variables. A logistic regression model was used to identify the variables that were most strongly associated with the level of need.

**Results:**

In 2010, the prevalence of the need for dental care in the SIRS cohort was 35.0% (95% CI [32.3–37.8]). It was lower in people with higher education levels (31.3% [27.9–34.6]), without immigrant background (31.3% [28.0–34.6]) and with comprehensive health insurance (social security + complementary health cover; 32.8% [30.2–35.4]). It decreased as the socio-economic status increased, but without following a strict linear change. It was also lower among individuals who had a dental check-up visit in the previous two years. In multivariate analyses, the socioeconomic variables most strongly associated with the need for dental care were: educational attainment (OR = 1.21 [1.02–1.44]), income level (OR = 1.66 [1.92–2.12]) and national origin (OR = 1.53 [1.26–1.86]).

**Conclusion:**

These results confirm that the prevalence of dental care needs is higher among adults with low socio-economic status. Education level, income level and also national origin were more strongly associated with the need for dental care than insurance cover level.

## Introduction

In 2004, a national oral health prevention plan was included in the French Public Health Law for the first time. Its purpose was to develop prevention policies among high-risk groups (children, disabled people, functionally dependent elderly and pregnant women), to improve dental care use and to prevent oral cancer [1]. Since then, the French Directorate for Research, Studies, Evaluation and Statistics (DREES) collects yearly data on especially developed indicators to monitor the implementation of the objectives of this law.

In the past decades, oral health has improved. Better diet and better oral hygiene and the widespread use of fluoridated products, such as water, salt or toothpastes, have been proposed as factors that have contributed to this significant improvement [2, 3, 4, 5, 6]. However, oral health inequalities based on socio-economic characteristics such as educational level, occupational background, income [6, 7, 8, 9, 10, 11, 12] and place of residence [13, 14] have been identified in many countries. But we do not know if these results are transposable to French population. Indeed in France, the impact of the socio-economic factors is strongly alleviated by the social transfers. In France, the social transfers lead to a 41% reduction of poverty and a 50% reduction of the disparities between the 20% wealthiest and the 20% poorest. Additionally, the amount to be paid by the patient after reimbursement by the health insurance is one of the lowest in Europe [15].

Most of the french studies on oral health and individual socio-economic determinants have focused on children. Studies regarding French adult population are very scarce and did not include representative samples. Thus at least 1 decayed tooth needing treatment was found in 33% of the 35–44 years old adults attending a dental consultation in medical centers of the national health insurance between 1999 and 2004 [16]. In another study, at least one untreated decayed tooth was found in 40% of the in the 30–50 years old adults attending a dental consultation in the farmers insurance dental centers (farmers, response rate to dental visit invitation = 22%) [17].

Therefore representative epidemiological data on the adult population in France are missing, as stressed also by the French National Authority for Health (Haute Autorité de Santé, HAS) in its 2010 data review and recommendations [18].

In this study we evaluated the prevalence of self-reported need for dental care in a representative sample of the adult population of the Paris region.

The tested hypothesis was that in adult French population socio-demographic and individual factors were associated with the need for dental care.

## Material and Methods

### Data sources

The SIRS (French acronym for “Health, Inequalities and Social Ruptures”) cohort study aims at studying the individual and contextual determinants of health [19]. The cohort population is representative of French-speaking adults living in Paris and its suburbs. In 2005, 3,000 people were randomly selected using a three-stage stratified sampling method (50 districts, 60 housing units per district and one adult per housing unit using the birthday method) (refusal rate of 22%). In 2010, participants were contacted again to carry out a new survey. Among the adults enrolled in 2005, 47% could be interviewed again. Indeed, 2.6% had died, 1.8% were too sick to participate, 13.9% had moved out of the Paris area, 2.7% were absent during the survey, 18.4% refused to answer and 13.4% could not be contacted. Overall, the sex ratio and mean age of people who could be interviewed again and those who could not were comparable. However, lost to follow-up people were more affluent than the others, but their type of place of residence and their health status were not different. Conversely, people absent at the time of the survey had a lower socio-economic status and were more often immigrants. People who were included in 2005 and who could not be re-interviewed in 2010 were replaced in the 50 districts of the cohort by using the same sampling method (refusal rate: 29%).

In order to be representative of the Parisian adult population, the sample has been adjusted (survey weight taking into account the over representation of underprivileged areas and the household size). The adjusted sample has been standardized by age and gender on the 2006 census basis.

To carry out a cross-sectional analysis of the stated need for dental care we used the data collected in the 2010 face-to-face survey during which people had to answer the question "Do you have one or more teeth that need to be treated, in poor condition, or to be replaced?” (1 among 370 questions).

We then analyzed the self-reported need for dental care relative to the demographic (gender, age, origin [French: both parents are French; French with immigrant background: at least one foreign parent; Foreigners]) and socio-economic characteristics (socio-professional group, average monthly income per consumption unit), educational level (lower secondary education; high school diploma; bachelor degree) and type of health insurance cover (Table 1).

**Table 1:** Prevalence of the declared need for oral care according to the characteristics of the study population (French SIRS cohort, 2010).

	**nb**	**Prevalence of oral care needs**		
		**%**	**p**	**[95% CI]**
**Total**	2977	35.06		[32.28–37.84]
**Gender**				
Men	1172	36.99		[32.72–41.25]
Women	1805	33.37	0.14	[30.24–36.48]
**Age**				
18–29 y/o	372	33.08		[25.75–40.41]
30–44 y/o	866	37.85		[33.22–42.49]
45–59 y/o	832	37.49		[33.82–41.15]
60 y/o and older	907	30.61	0.12	[27.10–34.12]
**Origin**				
French	1984	31.30		[28.02–34.58]
French with an immigrant background	603	43.16		[38.19–48.13]
Foreigners	390	41.62	10^−3^	[32.73–50.51]
**Education level**				
Bachelor degree	1433	31.25		[27.92–34.57]
High school diploma	638	41.65		[36.25–47.06]
Lower secondary education	906	38.65	<10^−3^	[34.66–42.64]
**Socio-professional group**				
Manager, intellectual profession	805	33.77		[26.42–35.11]
Intermediate profession	422	30.82		[26.11–35.54]
Craftsman, trader	143	30.47		[20.80–40.14]
Employee	1174	40.54		[36.33–44.75]
Worker	242	44.36		[36.41–52.32]
Has never worked	191	29.35	0,02	[17.23–41.46]
**Income per consumption unit**				
1^st^ quartile > 2,605	665	29.38		[24.77–33.99]
2^nd^ quartile >1,733 & ≤ 2,605	706	28.25		[23.77–32.74]
3^rd^ quartile > 1,115 & ≤ 1,733	759	38.25		[33.36–43.15]
4^th^ quartile ≤ 1,115	847	44.43	<10^−3^	[39.53–49.33]
**Health insurance**				
Social security + top-up cover	2427	32.77		[30.15–35.39]
CMU + CMU-C	207	44.68		[34.54–54.84]
CMU or social security alone	325	45.81		[39.52–52.10]
Don’t know/No health coverage	17	47.12	<10^−3^	[19.47–74.76]
**Last dental check-up**				
Less than 2 years ago	2297	33.70		[30.70–36.71]
More than 2 years ago	680	39.93	0.02	[35.01–44.87]

CMU: universal healthcare cover; CMU-C: CMU + free complementary cover.

In France, people are usually affiliated to the national health insurance (social security) through their direct (employees and self-employed) and indirect (employer) contributions to the social security system. The national health insurance normally covers about 70% of the general practitioner’s fees and 65% of the prescribed drugs but only 20% of dental prosthetics (for example a full metal crown costs around 300€ and the health assurance reimburses only 75.25€; or a 7 teeth metal removable denture costs around 1000€ and the health assurance reimburses only 120.40€). For additional reimbursement of the healthcare costs, people need to have a complementary health insurance (‘top-up’ cover).

People who are not covered by the national health insurance are entitled to the “Couverture Maladie Universelle” (CMU; Universal Healthcare Cover). It is free of charge for people with a low income (<9,534 Euros per household/annum) and above this threshold, they contribute 8% of their net income. To people on low income, the CMU offers a free complementary health cover (CMU-C) whereby medical and dental care are provided free of charge.

We thus classified participants in four categories, according to their type of healthcare coverage: i) social security + top-up cover; ii) CMU + CMU-C; iii) CMU alone or social security alone; iv) no health care coverage.

To evaluate their attitude and behavior regarding the prevention of oral diseases and dental problems, we also identified the participants who reported having had a dental check-up visit (in the absence of symptoms) in the previous two years. Although guidelines recommend an annual check-up visit, we considered that a visit in the previous two years was an acceptable preventive behavior.

### Statistical analysis:

We estimated the prevalence of the self-reported dental care need relative to each of the variables described above.

For each variable, we tested the association with the need for dental care and calculated the level of significance.

We then applied a logistic regression model to the unweighted data (adjusted for age and sex) in order to identify variables associated with the need for dental care. To identify the most strongly associated variables, we constructed an initial model that included all variables with a significance level lower than 0.20 in the previous prevalence analysis. The final model retained the most strongly associated variables after a step-by-step backward selection procedure. We set the type I error to 0.05. We performed all statistical analyses with StataCorp version 11.

This cohort study was authorized by two French national ethics committees for non-biomedical research: the *Comité consultatif sur le traitement de l’information en matière de recherche dans le domaine de la santé* (CCTIRS) and *the Commission nationale de l’informatique et des libertés* (CNIL).

## Results

Among the 3,006 people who were interviewed in the face-to-face survey of 2010, 29 participants did not answer the question about their dental care needs and therefore we conducted the analysis on a sample of 2,977 people (Table 1).

The prevalence of dental care needs in the whole population was 35.1% (95% CI [32.3–37.8]). This result did not vary significantly when participants were divided by gender and by age, although the observed prevalence was higher for men (37.0%) than for women (33.4%) and among the 30–59 age group (37.5%-37.8%) (Table 1).

Conversely, the need for dental care was significantly different in the three origin-based groups with the lowest prevalence observed in the French group (31.3% [28.0–34.6], p <10^−3^). Concerning the socio-economic characteristics, prevalence tended to decrease as the socio-economic status increased, without following a strict linear change. The “worker” and “employee” categories presented the highest prevalence of dental care needs. Similarly, the prevalence of dental care needs was significantly higher in the two less educated groups (38.6% and 41.6% compared to 31.25%, p <10^−3^). People who had both the social security and top-up health cover (81.5% of the cohort) reported fewer dental care needs (32.8% CI [30.1–35.4]) than the other three groups (p <10^−3^). Finally, the prevalence of the need for dental care was higher among people who did not have a dental check-up visit or dental plaque removal in the previous two years (39.9% versus 33.7%, p = 0.02).

The odds ratios (OR) obtained from the logistic regression "initial model" (Table 2, column 1) indicated how strong was the association between the need for dental care and individual characteristics adjusted for age and sex. These results confirmed that the need for dental care was higher in the lower educational and socio-economic categories. Specifically, a low level of education was associated with a 55% increase in the need of dental care (OR = 1.55 [1.33–1.80]). The strongest association was with the income level, as indicated by the finding that the prevalence of dental care needs in the least affluent quartile was twice that of the wealthiest quartile (OR = 2.10 [1.69–2.62]). Among the participants affiliated to the CMU-C the need for dental care was about 68% higher than in people with both social security and top-up health cover (OR = 1.68 [1.26–2.24]). Subjects without any health insurance coverage or who could not answer this question were far too few (n = 18) to draw conclusions. Finally, in people who did not have a dental check-up in the previous two years, the need for dental care was increased by about 31% (OR = 1.31 [1.10–1.56]).

**Table 2:** Association between the need for oral care and individual socio-demographic characteristics (French SIRS cohort, 2010).

	Initial model*			Final model**		
	OR	[95% CI]	p	OR	[95%CI]	p
Education level***						
Bachelor degree	ref	-	-	-	-	-
Up to high school diploma	1.55	[1.33–1.80]	<10^−3^	1.21	[1.02–1.44]	0.03
**Origin**						
French	-	-	-	-	-	-
French with an immigrant background	1.80	[1.46–2.12]	<10^−3^	1.53	[1.26–1.86]	<10^−3^
mmigrants	1.60	[1.27–1.98]	<10^−3^	1.19	[1.01–1.62]	0.04
**Socio-professional group**						
Manager, intellectual profession	ref	-	-	-	-	-
Intermediate profession	1.08	[0.83–1.39]	0.55	-	-	-
Craftsman, trader	1.13	[0.77–1.65]	0.52	-	-	-
Employee	1.74	[1.44–2.12]	<10^−3^	-	-	-
Worker	1.80	[1.34–2.43]	<10^−3^	-	-	-
Has never worked	1.43	[0.99–2.03]	0.05	-	-	-
**Income per consumption unit**						
1^st^ quartile > 2,605	ref	-	-	-	-	-
2^nd^ quartile >1,733 & ≤ 2,605	0.97	[0.77–1.22]	0,83	0.92	[0.72–1.16]	0.45
3^rd^ quartile > 1,115 & ≤ 1,733	1.62	[1.30–2.03]	<10^−3^	1.42	[1.12–1.80]	0.004
4^th^ quartile ≤ 1,115	2.10	[1.69–2.62]	<10^−3^	1.66	[1.92–2.12]	<10^−3^
**Medical coverage**						
Social security + top-up cover	ref	-	-	-	-	-
CMU + CMU-C	1.68	[1.26–2.24]	<10^−3^	-	-	-
CMU alone or social security alone	1.54	[1.22–1.95]	<10^−3^	-	-	-
Don’t know/No health coverage	1.97	[0.75–5.12]	0,17	-	-	-
**Last dental check-up**						
Less than 2 years ago	ref	-	-	-	-	-
More than 2 years ago	1.31	[1.10–1.56]	0,002	-	-	-

CMU: universal healthcare cover; CMU-C: CMU + free complementary cover

* model including each individual variable with adjustment for age and gender.

** model including age and gender and the significant variables with a cut-off set at 0.05% and after a stepwise regression analysis.

***education was grouped into two classes because the prevalence of dental care needs among people with high school diploma and those with lower secondary education was very similar (Table 1).

After the stepwise regression analysis (Final model, Table 2), only education attainment, origin and income level were retained as significantly associated with the need for dental care. The strongest link was between income level and self-reported need for dental care (OR fourth quartile versus first quartile = 1.66 [1.92–2.12]). The health cover was not anymore significantly associated with the prevalence of the declared need for dental care. This may be due to multi-collinearity. Indeed socio-economic variable were highly correlated themselves (Table 3): having no full medical cover was highly associated with 1) low income (OR = 8.84), 2) immigrant origin (OR = 4.64) 3) up to high school education level (OR = 2.32). The type of cover is a weaker determinant than income, origin or education level.

**Table 3:** Association between the medical cover and the individual socio-demographic characteristics model including each individual variable with adjustment for age and gender. (French SIRS cohort, 2010).

** **	Initial model*		
	**OR**	**[95% CI]**	**p**
**Education level**			
Bachelor degree	Ref	-	
Up to high school diploma	2.32	[1.82–2.95]	<10^−3^
**Origin**			
French	ref	-	
French with an immigrant background	2.98	[2.28–3.90]	
Immigrants	4.64	[3.49–6.16]	<10^−3^
**Income per consumption unit**			
1^st^ quartile > 2,605	Ref	-	
2^nd^ quartile >1,733 & ≤ 2,605	1.75	[1.03–2.98]	
3^rd^ quartile > 1,115 & ≤ 1,733	3.74	[2.30–6.07]	
4^th^ quartile ≤ 1,115	8.84	[5.58–14.03]	<10^−3^

* model including each individual variable with adjustment for age and gender.

The medical cover was dichotomized into 2 modalities: 1) Social security + top-up cover, “CMU + CMU-C” 2) “CMU alone or social security alone” or “Don’t know/No health coverage”.

## Discussion

This study shows that in a representative sample of adults from the Paris area (Paris city and suburb) the prevalence of self-reported need for dental care is of about 35.0%. This result seems to be in accordance with previous studies where the proportion of adults with at least one tooth to be treated was 33% and 40% [16, 17]. But the comparison should be cautious because the methods, populations and recorded variables were not the same (not representative samples, clinical examination, only decayed teeth).

Our study also identified significant associations between the prevalence of need for dental care and individual socio-economic characteristics, particularly the education attainment, territorial origin and income level.

In France, the link between oral health and socio-economic characteristics has been previously shown in studies carried out in children [2, 20]. In these works, the level of oral health in children, as reflected by the decayed, missing, filled teeth (DMFT) index [20] or the presence of periodontal disease or dental trauma [2], was inversely correlated with the parents’ socio-economic status and living conditions. In our study the association between education level and dental care needs was strongly significant. Education might lead to increase the importance people attach to their health and particularly to their oral health [2, 9]. Education might also facilitate the understanding of the mechanisms underlying disease development and the need to implement preventative measures and treatment compliance [21].

Our study also identified a higher need for dental care in participants with an immigrant background. The SIRS cohort included only French-speaking people; consequently the observed differences cannot be ascribed to communication difficulties. The French Institute for Research and Documentation in Health Economics (IRDES) also showed that the level of general health is worse among immigrants [22] and the SIRS cohort data enabled to identify origin-related inequalities not only in healthcare but also in the participation in health screening programs [23, 24]. To our knowledge, no study has been carried out specifically on the oral health of immigrants in France. However, several North American and European studies have revealed that oral health is poorer among immigrants, regardless of the health system and health coverage [2, 25, 26]. Several determinants were hypothesized, such as the language barrier that might limit the access to services, difficulties in diagnosis, deferrals or denial of care and loss of social ties [22].

In our study, the income level also was strongly associated with the need for dental care. Indeed, the prevalence of self-reported dental problems was twice higher when the income was lower than 1,115 Euros per month, compared to the prevalence among participants with an income of 2,605 Euros or higher. This finding is consistent with the previous analysis of the SIRS cohort where financial problems were given as a reason of renouncement to dental care by 10.4% of this population [27]. Likewise, in the Health and Social Protection Survey (ESPS) of 2004 conducted among a French population of 8,000 households, almost half of the insured had forgone or postponed dental, prosthetic and orthodontic care for financial reasons [28]. Low income was also previously found to be associated with a higher number of missing teeth [29].

In the current study, participants with a complementary health cover reported fewer instances of unmet care. This is in agreement with the French ESPS survey of 2000 showing that the consumption of dental care was higher and the likelihood to forgo dental care was lower in people with a complementary health insurance [7]. A complementary health coverage seems therefore to favor the access to dental care, including prosthetic care, and this improvement is particular evident in low-income individuals [7, 30]. Many studies have confirmed the remedial effect of a good level of health coverage on health inequalities [26, 31, 32].

But our results suggest that education and income level are more powerful determinants. Economic measures alone (for instance, the CUM-C) might not be sufficient to reduce the social inequalities in oral health. The authors proposed several possible explanations: social vulnerability which could lead to relegate the need for dental care to a lower level of priority, the ignorance of the health and social protection system (including the coverage of dental treatments by the CMU-C), but also the refusal of some practitioners to treat CMU-C beneficiaries [21, 33, 34].

If confirmed, this result emphasizes that policies based only on improving affordability are insufficient to completely reduce social inequalities in oral health [35, 36, 37, 38].

A low prevalence of dental care needs was observed in individuals who underwent a dental check-up in the previous two years. Regular visits might decrease the amount of unmet needs. Concomitantly, the absence of symptoms and of important needs can be an incentive to consult without fearing dental care, which is sometimes thought to be costly or painful. Indeed, studies about healthcare renouncement also suggest that people with the greatest needs are also those more likely to renounce [7, 39].

Our study did not highlight any significant gender-related differences. However, according to the 2000 ESPS, women have a better oral health status than men, possibly because they are more conscious about oral health and have more regular dental check-up visits [7]. Similarly, a recent study in four industrialized countries showed a significant influence of age on the need for oral care [40].

One limitation of this study is that our results are based on the self-reported need for dental care. A measurement based on a clinical examination would have been more objective and accurate. Indeed the self-reported dental need can introduce a bias in two different ways: 1) a person has a worse oral condition than declared; 2) even though objective dental condition is the same, a person perceives his/her dental need more important compared to others. It could be asked whether, how and to what extent the "self-reporting" factor has affected the results described in this study. It was shown that self-reporting underestimated the oral care needs [41, 42] but that it was a relatively valid indicator. Indeed self-reports have been shown [42] to be strongly correlated (r = 0.74–1.0) to the numbers of remaining teeth, fillings, root canal therapy, and prostheses. However, they appeared to be less accurate for the assessment of dental caries and periodontal disease (r = 0.47–0.56). This study [42] found a high sensitivity (90.0%–100.0%) for these items, except for dental caries (moderate sensitivity 59.5%) and periodontitis (low sensitivity 39.3%). The positive predictive values of all measures were high, ranging from 66.7% to 100.0% [42]. Thus real oral condition is probably worse than reported in this study.

Regarding general and oral health, studies in which self-reported and clinical data were collected and compared suggest that individuals with low socio-economic status tend to underestimate their level of healthcare requirements [33, 34, 41, 43, 44, 45]. If this occurred also within the SIRS cohort population, our results would have underestimated the importance of the socio-economic gradient.

The cross-sectional nature of our analysis should also be taken into account as it does not allow interpreting the identified associations as causal links.

Finally, although the results of the analysis of the need for dental care in the SIRS cohort are consistent with other French national surveys [7, 46], caution should be applied in their generalization because of the specificity of the Paris population (younger, with more skilled jobs and a mean income level above the French national average).

In conclusion, our study suggests that despite improvements of the oral health status in the adult population, significant disparities still exist particularly among the less educated, with low level of resource and immigrant people. Although beneficial, the economic measures (for instance, the CUM-C) aiming at facilitating the access/use of dental care might not be sufficient to reduce the social inequalities in oral health. Other complementary determinants need to be considered and further investigated, particularly the absence/presence of social ties and the access to healthcare facilitation services.

The role of local associations in maintaining/encouraging social interactions and in orienting toward dental care and administrative services has to be supported. [47]

The French law of 2008 on hospitals, patients, health and territories (HPST law) implemented some measures to improve health care access in general and dental medicine. Their impact will have to be assessed.

## Supporting Information

S1 File Base_individus_Plos_One.dta. Study data from SIRS Cohort.
(DTA)
